# Association Analysis Between Dietary Magnesium Intake and All‐Cause and Cause‐Specific Mortality in Individuals With Chronic Neck Pain

**DOI:** 10.1002/mnfr.70503

**Published:** 2026-05-17

**Authors:** Renkun Zhao, Dongsheng Yuan, Penghui Li, Peng Zhang, Jiaming Wei, Guoliang Ma, Zhizhuang Wang, Bo Xu, Xiaokuan Qin, Bowen Yang, Liguo Zhu, He Yin, Kexin Yang, Xin Chen

**Affiliations:** ^1^ Department of Spine Wangjing Hospital of China Academy of Chinese Medical Sciences Beijing China; ^2^ Hunan University of Chinese Medicine Changsha China; ^3^ Department of Knee Joint Luoyang Orthopedic Hospital of Henan Province (Henan Orthopedic Hospital) Luoyang China

**Keywords:** all‐cause mortality, cancer mortality, cardiovascular disease mortality, chronic neck pain, C‐reactive protein, dietary magnesium, inflammation

## Abstract

This study investigated the association between dietary magnesium intake and all‑cause and cause‑specific mortality among individuals with chronic neck pain (CNP). We enrolled 2955 CNP patients aged ≥20 years from the 1999–2020 NHANES database and used Cox regression, subgroup analysis, and Mendelian randomization‑based mediation analysis to explore relevant associations and mechanisms. During follow‑up, 719 all‑cause deaths, 180 cardiovascular disease (CVD) deaths, and 179 cancer deaths occurred. Multivariable‑adjusted analyses showed that higher dietary magnesium intake was significantly associated with reduced CVD mortality, with each 1 g/day increment linked to a 78% lower risk. A non‑linear association with a threshold of 0.5181 g/day was found for cancer mortality, above which mortality risk dropped significantly. Subgroup analyses confirmed result robustness. Mediation analysis revealed that magnesium lowered all‑cause and CVD mortality directly and indirectly by reducing C‑reactive protein (CRP) levels. These findings indicate that higher dietary magnesium intake predicts lower mortality risk in CNP patients, partly via CRP‑mediated anti‑inflammatory pathways, supporting dietary magnesium optimization from whole‑food sources to improve long‑term prognosis.

## Graphical abstract

Higher dietary magnesium intake is associated with lower risks of all‐cause, cardiovascular disease, and cancer mortality in individuals with chronic neck pain. The beneficial effects are partially mediated by reduced C‐reactive protein (CRP) levels. Optimizing dietary magnesium may improve long‐term prognosis in this population.

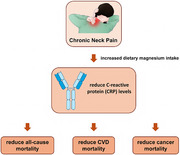

## 1 Introduction

Chronic neck pain (CNP) is defined as neck pain lasting more than 3 months due to cervical spondylosis [1], which typically presents radiologically as loss of cervical lordosis and degenerative changes including facet joint hyperplasia [2]. Clinically, it is characterized by limited cervical motion, stiffness, upper limb soreness and numbness, hand weakness, and muscle spasms. Affected individuals often experience concurrent emotional disturbances such as anxiety, depression, and low mood. For this study, CNP was strictly defined using standardized questionnaire items from the NHANES database, consistent with the inclusion criteria detailed in the Methods section. This condition not only impairs the quality of life of affected individuals but also imposes a substantial socioeconomic burden.

Findings from Hirai T et alet al. [3]. suggest that CNP may be associated with cervical dysfunction (e.g., spinal cord disorders), leading to gait instability, impaired balance, and significantly elevated fall risk—a leading cause of mortality in older adults. Identifying biomarkers that predict and reduce adverse outcomes in individuals with CNP is therefore of considerable clinical importance. Previous studies indicate that genetic factors, obesity, and unhealthy dietary behaviors may increase the incidence or severity of CNP [4].

Magnesium is an essential trace element involved in maintaining metabolic balance and energy regulation. Moreover, a growing body of research suggests that dietary magnesium intake is closely linked to the development of CNP. Studies by Okada A et al. [5]. and Bagheri R G et al. [6]. demonstrated that magnesium, acting as a natural calcium‐channel blocker, promotes calcium efflux from cells and effectively relieves muscle tension and spasms in individuals with CNP. Meanwhile, magnesium exerts strong anti‐inflammatory effects by inhibiting pathways such as nuclear translocation of nuclear factor‐κB (NF‑κB), lowering levels of pro‐inflammatory cytokines including tumor necrosis factor‐α (TNF‑α), C‐reactive protein (CRP), and interleukin‐6 (IL‑6), and slowing cervical degeneration, thereby further reducing neck pain. These findings indicate that higher magnesium intake may help prevent and relieve CNP.

However, these findings are contradicted by a study by Igwe AA et al. [7], which found no significant association between serum magnesium levels and CNP severity. Prospective evidence indicates that increased magnesium intake may reduce pressure on respiratory control centers and prevent respiratory depression by alleviating severe cervical dysfunction (e.g., spinal cord compression) associated with CNP. Nevertheless, higher magnesium intake was not associated with a significant reduction in the risk of CNP onset or progression [8]. In cellular experiments, Lian X et al. [9]. reported that magnesium sheets modified with dexamethasone‐loaded DNA hydrogel used as a barrier membrane directly reduced neck pain by suppressing the expression of pro‐inflammatory factors IL‑1β and TNF‑α in mouse macrophages (RAW264.7). Magnesium also inhibits osteoblast differentiation pathways induced by osteogenic signals such as TGF‑β, which delays osteophyte formation and addresses the underlying triggers of CNP. However, further investigation is needed to clarify the precise mechanisms. In addition, previous research has demonstrated strong associations between magnesium intake and several chronic conditions, including type 2 diabetes and hypertension [10, 11]. Two cohort studies have also reported an inverse association between magnesium intake and all‐cause mortality [12, 13].

Further research is warranted because conclusive evidence regarding the association between dietary magnesium intake and cause‐specific or all‐cause mortality among individuals with CNP remains lacking. Identifying optimal magnesium intake levels is particularly critical for individuals with CNP, as dietary requirements may vary across populations based on factors including sex, age, and body composition. This study aimed to investigate the association between dietary magnesium intake and both cause‐specific and all‐cause mortality among individuals with CNP using data from the National Health and Nutrition Examination Survey (NHANES). The objectives were to provide evidence‐based support for improving the prognosis of CNP through dietary interventions and to propose appropriate dietary magnesium intake guidelines for diverse subgroups.

## 2 Material and Methods

### 2.1 Data Origins

This retrospective cohort analysis used the 1999–2020 NHANES database, maintained by the National Center for Health Statistics (NCHS) at the U.S. Centers for Disease Control and Prevention (CDC), which is designed to assess the nutritional and health status of the civilian U.S. population [14]. NHANES collects data from a representative sample of non‐institutionalized individuals using a multistage, probability‐based sampling design, ensuring the sample is nationally representative of the U.S. population. The survey is conducted biennially [15]. The NHANES protocol was approved by the NCHS Ethics Review Committee [16]. All participants provided written informed consent before enrollment. Details regarding the official NHANES study design and data access are available at www.cdc.gov/nchs/nhanes/. The main data sources for NHANES include household interviews and self‐reported questionnaires, which may be subject to recall bias.

### 2.2 Methods

#### 2.2.1 Assessment of Dietary Magnesium Intake and Diagnosis of CNP

National Health and Nutrition Examination Survey (NHANES) employed a 2‐day 24‐hour dietary recall method to assess magnesium intake; the first interview was conducted in person, and the second via telephone. Dietary magnesium intake was calculated as the average of the two 24 h dietary recall measurements. Nutritional values were estimated using the U.S. Department of Agriculture (USDA) Food and Nutrition Database for Dietary Reference Values, and participants provided detailed records of all food and beverage consumption over the preceding 24 h [17].

Per the NHANES protocol, proxy respondents were permitted to assist with dietary recall interviews for participants with cognitive impairment, dementia, or severe memory loss. Individuals with severe cognitive dysfunction who could not provide reliable dietary data even with proxy assistance were excluded from the final analysis.

Since NHANES is a population‐based observational survey and does not collect formal clinical diagnostic codes (ICD‐9/10) for CNP, CNP was strictly defined in this study using specific questionnaire items from the NHANES database to ensure the consistency and reproducibility of the study population. The exact survey question used was: “Have you had neck pain, soreness, or stiffness for 3 months or longer?” (corresponding to NHANES questionnaire codes ARQ024A and MPQ060). Participants who responded “Yes” to this question were classified as having CNP, while those who responded “No” were excluded from the CNP cohort.

#### 2.2.2 Determination of Mortality

Mortality data for each participant was linked to the National Death Index (NDI) through December 31, 2024. Causes of death were identified using the International Classification of Diseases, 10th Revision (ICD‐10) codes [18]. The primary study outcomes included all‐cause mortality, CVD‐related mortality (ICD‐10 codes I00–I09, I11, I13, I20–I51), cancer‐related mortality (ICD‐10 codes C00–C97), and mortality from other causes. Cancer mortality was determined exclusively based on death certificate codes (ICD‐10 C00–C97) from the NDI. No additional cancer screening or diagnostic evaluations were conducted at baseline, as this was a population‐based observational cohort study that relied on linked mortality data.

#### 2.2.3 Selection of Covariates

Covariate selection was guided by criteria from previous studies and theoretical considerations (e.g., selection bias arising from significant missing covariate data) [19]. Covariates included gender (male/female), age, race (Mexican American/other Hispanic/non‐Hispanic White/non‐Hispanic Black/other), alcohol consumption (≥4 drinks/day), smoking status (ever smoked ≥100 cigarettes), household income‐to‐poverty ratio (0–1/>1 and <3.5/≥3.5), body mass index (BMI), hypertension, diabetes, and physical activity. BMI was categorized into three groups: normal (18.5–24.9 kg/m^2^), overweight (25–30 kg/m^2^), and obese (>30 kg/m^2^). Diabetes was diagnosed based on either self‐reported physician diagnosis or adherence to the U.S. CDC criteria, which define undiagnosed diabetes as a glycated hemoglobin level ≥6.5% or a fasting blood glucose level ≥7.0 mmol/L [20]. Hypertension was diagnosed based on self‐reported physician diagnosis or blood pressure measurements meeting the CDC threshold (systolic blood pressure ≥140 mmHg and/or diastolic blood pressure ≥90 mmHg) [21]. Physical activity was quantified using metabolic equivalent tasks (METs), calculated as the number of weekly active days multiplied by the MET value and duration (in minutes) of each activity [22]. Comprehensive measurement guidelines for all variables are available on the official NHANES website.

As a key mediator in the mediation analysis, serum CRP concentrations were measured using a high‐sensitivity immunoturbidimetric assay in the NHANES laboratory. Venous blood samples were collected from participants following an overnight fast of at least 8 h, and all assays were performed in accordance with standardized quality control protocols established by the NCHS.

#### 2.2.4 Study Sample

**FIGURE 1:**
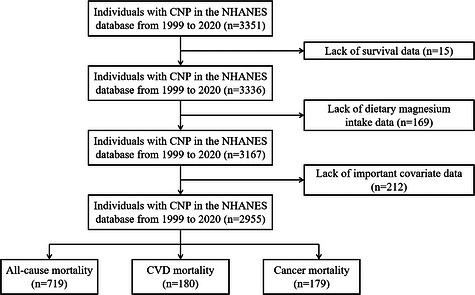
Individuals with CNP selection flowchart.

We included individuals aged ≥20 years with a diagnosis of CNP from the 1999–2020 NHANES database. Participants were excluded if they had missing survival data, missing dietary magnesium intake information, or missing data on key confounders (including diabetes, hypertension, smoking status, alcohol consumption, household income‐to‐poverty ratio, and educational attainment). A total of 3351 individuals with CNP were initially identified. After excluding 15 participants with missing survival data, 169 with missing dietary magnesium intake data, and 212 with missing critical covariate data, 2955 participants were ultimately included in the analysis. The participant screening process is illustrated in Figure 1.

#### 2.2.5 Statistical Analysis

All statistical analyses were performed using R 4.5.1 and SPSS 27.0 software, with *p* < 0.05 considered statistically significant. Chi‑squared tests were used for between‑group comparisons of categorical variables, and linear regression models were applied to assess associations for continuous variables. Results are presented as mean ± standard deviation for continuous variables and as frequencies and percentages for categorical variables. Three Cox proportional hazards regression models were constructed to evaluate the association between dietary magnesium intake and mortality: Model 1 was unadjusted; Model 2 was adjusted for age, gender, and race; and Model 3 was further adjusted for BMI, education level, household income‑to‑poverty ratio, hypertension, diabetes, smoking status, alcohol consumption, physical activity, and log‑transformed serum CRP. Nonlinear associations between dietary magnesium intake and mortality among individuals with CNP were explored using Cox models with smooth curve fitting and generalized additive models. Where nonlinear relationships were identified, threshold effect analysis was performed to detect potential inflection points in dietary magnesium intake. Two‑stage Cox regression models were then used to examine the association between magnesium intake and mortality on either side of these inflection points. Subgroup analyses and interaction tests were conducted with stratification by gender, age (<60 or ≥60 years), BMI (<25, 25–30, or ≥30), diabetes, hypertension, and physical activity. The mediating effect of CRP on the association between dietary magnesium intake and mortality in individuals with CNP was assessed using mediation analysis within a Mendelian randomization (MR) framework. Using genetic instrumental variables, we separately estimated the effect of the exposure (dietary magnesium intake) on the mediator (CRP), the effect of the mediator on the outcome (mortality), and the direct effect of the exposure on the outcome. This approach enabled us to evaluate whether CRP acts as a causal mediator in the pathway linking magnesium intake to mortality.

## 3 Results

### 3.1 Baseline Characteristics of Individuals with CNP

The average age of participants was 49.60 ± 16.61 years, with 31.39% being male and 68.61% female. According to Table 1, which details baseline characteristics stratified by quartiles of dietary magnesium intake, significant between‐group differences were observed in age, gender, race, education level, household income‐to‐poverty ratio, BMI, hypertension status, smoking status, alcohol consumption, and physical activity (*p* < 0.05). The recommended dietary allowance (RDA) for magnesium in adults is approximately 310–420 mg/day (0.310–0.420 g/day), as specified in U.S. dietary reference guidelines. Dietary magnesium intake was categorized into quartiles, where Q1 represented the lowest intake and Q4 the highest. The specific ranges of magnesium intake for each quartile were as follows: Q1 ≤ 0.1695 g/day, Q2 ≤ 0.242 g/day, Q3 ≤ 0.3297 g/day, and Q4 ≥ 0.3297 g/day. Participants with higher dietary magnesium intake had a significantly lower risk of hypertension compared with those in the lowest intake group (*p* < 0.001), whereas no significant difference was observed in the risk of diabetes between the two groups (*p* = 0.295). As this study used linked prospective mortality data and the baseline assessment was cross‐sectional, dietary magnesium intake was only evaluated at baseline and not reassessed during follow‐up. Therefore, longitudinal changes in magnesium intake and associated outcomes could not be tracked.

**TABLE 1:** Baseline characteristics of individuals with CNP included in the NHANES database.

	Total	Q1: ≤ 0.1695 g/d	Q2: 0.1696–0.242 g/d	Q3: 0.243–0.3297 g/d	Q4: > 0.3297 g/d	*p*‐value
Sample size	2955	736	743	737	739	
Age (X——*±S*, years)	49.60 ± 16.61	49.64 ± 16.43	51.57 ± 16.65	50.35 ± 16.07	46.85 ± 15.32	*P* < 0.001
Gender (*n*, %)						*P* < 0.001
Male	1249/42.27	231/31.39	245/32.97	321/43.55	452/61.16	
Female	1706/57.73	505/68.61	498/67.03	416/56.45	287/38.84	
Race (*n*, %)						*P* < 0.001
Hispanic	655/22.17	162/22.01	177/23.82	151/20.49	165/22.33	
Other Hispanic	185/6.26	54/7.34	51/6.86	37/5.02	43/5.82	
Non‐Hispanic White	1543/52.22	316/42.93	372/50.07	423/57.39	432/58.46	
Non‐Hispanic Black	475/16.07	175/23.78	123/16.55	101/13.70	76/10.28	
Other Ethnicities	97/3.28	29/3.94	20/2.7	25/3.40	23/3.11	
Education Level (*n*, %)						*P* < 0.001
Below high school	498/16.85	151/20.52	136/18.30	119/16.15	92/12.45	
High School	1247/42.20	336/45.65	329/44.28	304/41.25	278/37.62	
High school and above	1210/40.95	249/33.83	278/37.42	314/42.60	369/49.93	
Household Income‐to‐poverty Ratio (*n*, %)						*P* < 0.001
0‐1	838/28.36	259/35.19	226/30.42	179/24.29	174/23.55	
>1 and < 3.5	1315/44.50	329/44.70	340/45.76	325/44.10	321/43.44	
≥3.5	802/27.14	148/20.11	177/23.82	233/31.61	244/33.01	
BMI (X——*±S*, kg/m^2^)	28.69 ± 6.50	28.73 ± 6.91	28.90 ± 6.25	28.99 ± 6.77	28.17 ± 6.02	*p = 0.024*
Hypertension (*n*, %)						*P* < 0.001
No	1879/63.59	454/61.68	446/60.03	458/62.14	521/70.50	
Yes	1076/36.41	282/38.32	297/39.97	279/37.86	218/29.50	
Diabetes (*n*, %)						*P* = 0.295
No	2611/88.36	646/87.78	649/87.35	651/88.33	665/90.00	
Yes	344/11.64	90/12.22	94/12.65	86/11.67	74/10.00	
smoking (*n*, %)						*P* < 0.001
No	1355/45.85	646/87.77	649/87.35	333/45.18	329/44.52	
Yes	1600/54.15	90/12.23	94/12.65	404/54.82	410/55.48	
Drinking (*n*, %)						*P* = 0.001
No	2537/85.85	661/89.81	639/86.00	625/84.80	612/82.81	
Yes	418/14.15	75/10.19	104/14.00	112/15.20	127/17.19	
Physical Activity (*n*, %)						*P* < 0.001
No	1714/58.00	463/62.91	461/62.05	405/54.95	385/52.10	
Yes	1241/42.00	273/37.09	282/37.95	332/45.05	354/47.90	

*Note*: ① Q1–Q4 represent quartiles of dietary magnesium intake, with the following ranges: Q1: ≤ 0.1695 g/d; Q2: 0.1696–0.242 g/d; Q3: 0.243–0.3297 g/d; Q4: > 0.3297 g/d. ② “Other Hispanic” refers to Hispanic participants who are not of Mexican American origin, as defined by the NHANES race/ethnicity classification system. ③ For continuous variables (age, BMI), *p*‐values were calculated using one‐way analysis of variance (ANOVA). For categorical variables, P‐values were calculated using chi‐squared tests.

### 3.2 Relationship between Dietary Magnesium Intake and Mortality

Cox regression analysis (Table 2) demonstrated the association between dietary magnesium intake and mortality. When dietary magnesium intake was treated as a continuous variable, multivariate‐adjusted analyses showed that higher magnesium intake was significantly associated with a reduced risk of cardiovascular disease (CVD) mortality. Specifically, each 1 g/day increase in dietary magnesium intake was associated with a 78% reduction in the risk of CVD mortality (*p* = 0.015). Similar trends were observed for all‐cause mortality and cancer‐related mortality. Multivariate‐adjusted results (Model 3, fully adjusted for confounders) for mortality across quartiles of dietary magnesium intake were as follows: For all‐cause mortality: Q1 (reference group, *HR* = 1.00), Q2 (*HR* = 1.005, 95% *CI*: 0.826–1.222), Q3 (*HR* = 0.938, 95% *CI*: 0.762–1.155), Q4 (*HR* = 0.853, 95% *CI*: 0.679–1.072). For CVD mortality: Q1 (reference group, *HR* = 1.00), Q2 (*HR* = 0.749, 95% *CI*: 0.509–1.101), Q3 (*HR* = 0.770, 95% *CI*: 0.518–1.146), Q4 (*HR* = 0.495, 95% *CI*: 0.307–0.797). For cancer mortality: Q1 (reference group, *HR* = 1.00), Q2 (*HR* = 1.084, 95% *CI*: 0.721–1.632), Q3 (*HR* = 1.138, 95% *CI*: 0.751–1.726), Q4 (*HR* = 0.927, 95% *CI*: 0.583–1.474). Overall, compared with the lowest magnesium intake group (Q1), higher dietary magnesium intake was associated with a trend toward reduced risks of all‐cause, CVD, and cancer mortality in individuals with CNP. Notably, additional adjustment for log‐transformed CRP levels did not substantially weaken the inverse association between dietary magnesium intake and CVD mortality, indicating that the beneficial effects of magnesium may involve both anti‐inflammatory pathways and other metabolic mechanisms.

**TABLE 2:** Cox regression analysis of specific associations between dietary magnesium intake and mortality in individuals with CNP.

Variable	Model 1			Model 2			Model 3		
	*HR*	95%*CI*	*p*‐value	*HR*	95%*CI*	*p*‐value	*HR*	95%*CI*	*p*‐value
**All‐cause mortality**									
Dietary Magnesium Intake	0.326	0.189,0.564	< 0.001	0.404	0.228,0.718	0.002	0.570	0.323,1.003	0.051
Q1	1			1			1		
Q2	1.097	0.903,1.333	0.350	0.985	0.811,1.197	0.882	1.005	0.826,1.222	0.962
Q3	0.889	0.725,1.090	0.257	0.836	0.682,1.025	0.084	0.938	0.762,1.155	0.548
Q4	0.663	0.533,0.824	< 0.001	0.732	0.586,0.914	0.006	0.853	0.679,1.072	0.174
CVD mortality rate									
Dietary magnesium intake	0.166	0.053,0.521	0.002	0.162	0.049,0.537	0.003	0.220	0.065,0.741	0.015
Q1	1			1			1		
Q2	0.847	0.578,1.240	0.392	0.761	0.519,1.114	0.16	0.749	0.509,1.101	0.141
Q3	0.784	0.533,1.153	0.216	0.720	0.489,1.060	0.096	0.770	0.518,1.146	0.198
Q4	0.438	0.278,0.691	< 0.001	0.449	0.283,0.715	< 0.001	0.495	0.307,0.797	0.004
Cancer mortality rate									
Dietary magnesium intake	0.348	0.116,1.039	0.058	0.386	0.123,1.213	0.103	0.467	0.148,1.477	0.195
Q1	1			1			1		
Q2	1.225	0.816,1.838	0.328	1.112	0.741,1.669	0.610	1.084	0.721,1.632	0.697
Q3	1.142	0.758,1.719	0.526	1.064	0.706,1.604	0.767	1.138	0.751,1.726	0.542
Q4	0.803	0.516,1.251	0.333	0.849	0.540,1.335	0.479	0.927	0.583,1.474	0.750

*Note*: Model 1: Unadjusted; Model 2: Adjusted for age, gender, race; Model 3: Adjusted for age, gender, race, education level, household income‐to‐poverty ratio, BMI, hypertension, diabetes, smoking, drinking, physical activity; Magnesium intake Q1 ≤ 0.1695 g/day, 0.1695 g/day < magnesium intake Q2 ≤ 0.242 g/day, 0.242 g/day < magnesium intake Q3 ≤ 0.3297 g/day, magnesium intake Q4 > 0.3297 g/day.

### 3.3 Dose‐Response Relationship between Dietary Magnesium Intake and Mortality

**FIGURE 2:**
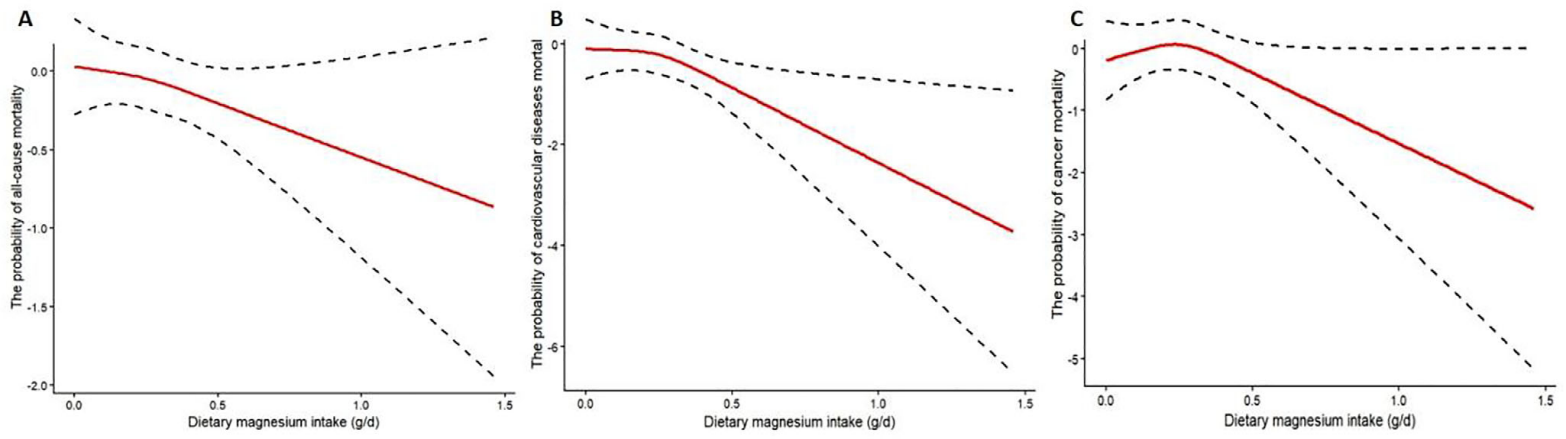
Association between dietary magnesium intake and all‐cause (A), CVD (B), and cancer (C) mortality in individuals with CNP. ***Notes***: Adjusted variables include age, gender, race, education level, household income‐to‐poverty ratio, BMI, hypertension, diabetes, smoking, drinking, and physical activity. Solid lines represent estimates; dashed lines indicate 95% CIs. P‐values for non‐linearity testing: all‐cause mortality, *p* = 0.994; CVD mortality, *p* = 0.595; cancer mortality, *p* = 0.173.

As shown in Figure 2 and Table 3, after full adjustment for confounding variables, generalized additive models and smoothing curve fitting further revealed a monotonically decreasing trend between dietary magnesium intake and mortality outcomes. Formal non‐linearity testing was performed by comparing linear Cox proportional hazards models with restricted cubic spline (RCS) models. The results showed P‐values of 0.994 for all‐cause mortality, 0.595 for CVD mortality, and 0.173 for cancer mortality, indicating no statistically significant non‐linear association for any of these outcomes. For cancer mortality, a trend toward a non‐linear dose‐response relationship was observed (log‐likelihood ratio, p = 0.01). Threshold effect analysis using a two‐stage Cox regression model— which identifies the turning point where the slope of the association changes significantly—was conducted to determine the inflection point. The inflection point was calculated to be 0.5181 g/day of dietary magnesium. Below this threshold, there was no statistically significant association between magnesium intake and cancer mortality. In contrast, above this threshold, a significant reduction in cancer mortality risk was observed (*HR* = 0.01), suggesting that a higher dietary magnesium intake (≥ 0.5181 g/day) may exert a protective effect against cancer mortality. Notably, no significant plateau effect was observed in the dose‐response curves across the tested range of magnesium intake. Importantly, we explicitly emphasize that the “more is better” principle does not apply here: the protective threshold of 0.5181 g/day identified in this study is well below the tolerable upper intake level (UL) of 0.70 g/day for adults. Excessive magnesium intake beyond this safe limit may increase the risk of hypermagnesemia, especially in individuals with impaired renal function.

**TABLE 3:** Threshold analysis of dietary magnesium intake on cancer mortality in individuals with CNP.

Variable		Adjusted *HR* (95% *CI*)	*p*‐value	Log‐Likelihood Ratio *p*‐value
Cancer Mortality				
Dietary Magnesium Intake	Standard linear model used to fit inflection point: 0.5181 g/d	0.467(0.148, 1.476)	0.194	0.01
	Fitted using a two‐segment linear model			
	Dietary magnesium intake < 0.5181 g/d	0.541(0.13,2.25)	0.398	
	Dietary magnesium intake ≥ 0.5181 g/d	1.37×10^−^^6^(4.12×10^−^^1^ ^3^, 4.58)	0.063	

### 3.4 Subgroup Analysis Results

**FIGURE 3:**
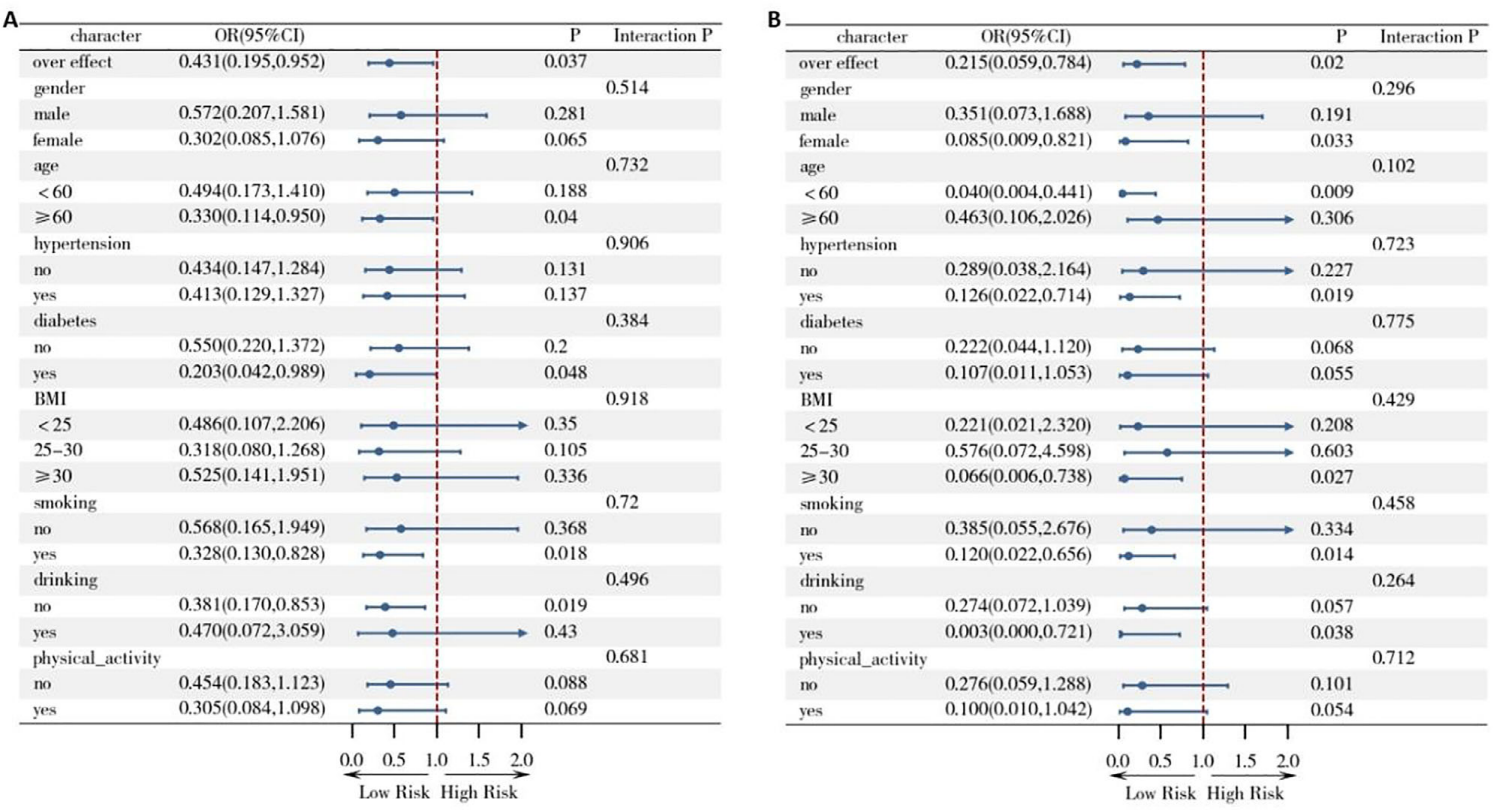
Subgroup analysis of dietary magnesium intake and all‐cause (A) and CVD (B) mortality in individuals with CNP. ***Notes***: Subgroup analysis variables included gender, age, hypertension, diabetes, BMI, smoking, drinking, and physical activity.

Subgroup analyses and interaction tests were conducted by stratifying participants according to age, gender, BMI, hypertension status, diabetes status, physical activity level, smoking status, and alcohol consumption. The purpose was to evaluate the consistency of associations across different populations and identify potential high‐risk groups. Stronger protective effects of higher magnesium intake on all‐cause mortality were observed in specific subgroups: participants aged ≥60 years, those with diabetes, ever smokers, and non‐drinkers (*p* < 0.05). For CVD mortality, more pronounced protective effects were observed in women, participants aged <60 years, those with hypertension, individuals with BMI ≥30 kg/m^2^, current smokers, and alcohol consumers (*p* < 0.05). Notably, no significant interactions were detected between dietary magnesium intake and the stratification factors (gender, age, comorbidities, and lifestyle) with respect to either all‐cause mortality or CVD mortality (Figure 3).

### 3.5 Mediating Effect Analysis Results

**FIGURE 4:**
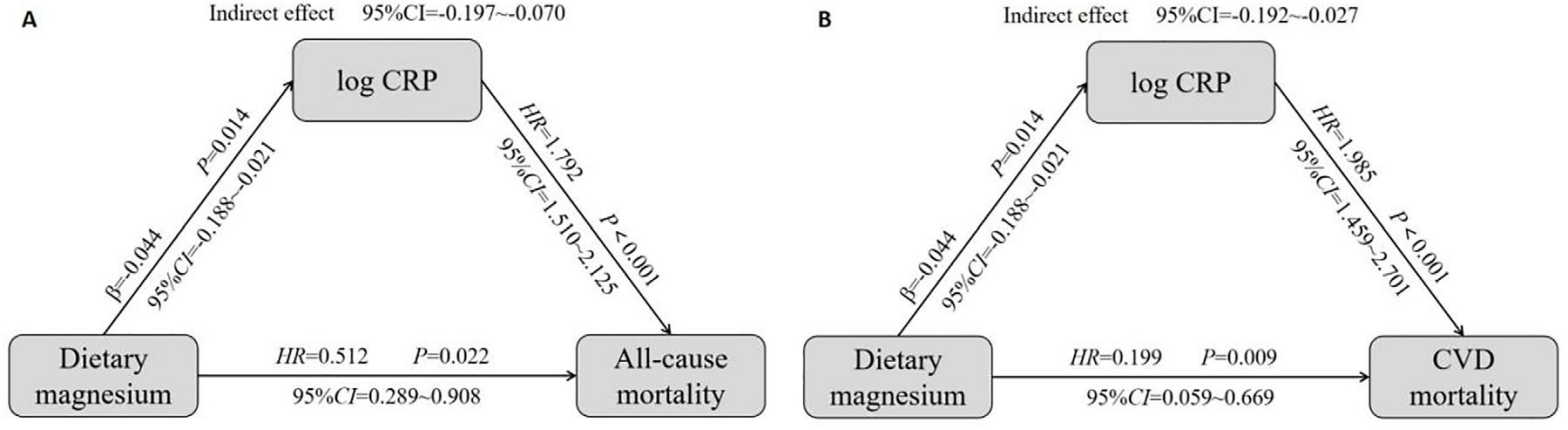
Mediating effects of dietary magnesium intake on all‐cause (A) and CVD (B) mortality in individuals with CNP. ***Notes***: Mediator analysis variables included gender, age, hypertension, diabetes, BMI, smoking, drinking, and physical activity. log CRP denotes the logarithmic form of CRP.

Given clinical evidence that CRP levels may simultaneously influence dietary magnesium intake and mortality risk in individuals with CNP, we further incorporated CRP into the multivariable model to explore its potential mediating role [23]. Due to the right‐skewed distribution of CRP levels, a log_10_ transformation was performed to enhance model fit and stability. We then evaluated the role of CRP as a mediator in the association between dietary magnesium intake and mortality in individuals with CNP. As illustrated in Figure 4, effect sizes were estimated using beta coefficients and inverse variance weighting. A significant negative association was observed between dietary magnesium intake and log‐transformed CRP levels (*β* = −0.044, 95% *CI* = −0.188 to −0.021, *p* = 0.014). In individuals with CNP, higher log CRP levels were significantly associated with increased all‐cause and CVD mortality (*p* < 0.001). The 95% CIs for the indirect effect did not include zero (95% *CI* = −0.197 to −0.070 for all‐cause mortality and −0.192 to −0.027 for CVD mortality), indicating a significant mediating effect of CRP. These findings suggest that dietary magnesium may indirectly reduce all‐cause and CVD mortality in individuals with CNP by lowering log CRP levels, which serves as a key mediating pathway. Additionally, dietary magnesium exerted a direct effect on both all‐cause and CVD mortality (*p* < 0.05). These results remained robust after adjusting for potential confounders, including age, gender, diabetes, and hypertension, further confirming the validity of the mediating role of CRP.

## 4 Discussion

The development of CNP is closely associated with cervical spine instability, which can present as an exogenous dynamic imbalance involving cervical muscles and soft tissues, as well as an endogenous static imbalance affecting the vertebrae, intervertebral discs, facet joints, and surrounding ligaments [24]. Previous studies have confirmed that pain induced by cervical instability is associated with the stimulation of nerve growth factor (NGF) production in cervical disc tissue by inflammatory mediators [25] and that inflammation is a key contributing factor to CNP [26]. These findings provide a pathological and physiological basis for future research on the role of dietary magnesium in CNP management.

Magnesium, an essential component of the human body, is involved in multiple cellular metabolic processes. Higher magnesium intake significantly reduces levels of inflammatory markers [27, 28], whereas inadequate magnesium intake is strongly associated with chronic inflammatory conditions [29, 30]. Theoretically, this property could help maintain the health of individuals with CNP by reducing the risk of mortality related to spinal cord compression or muscle atrophy, as well as slowing disease progression through the regulation of inflammatory responses. In light of this, participants with CNP in this study were categorized based on quartiles of dietary magnesium intake. Baseline characteristic analysis showed that the risk of hypertension in individuals with CNP significantly decreased with increasing dietary magnesium intake, while there was no significant difference in the risk of diabetes. Consistent with previous research, subgroup analysis revealed that compared with non‐hypertensive individuals with CNP, hypertensive individuals with CNP exhibited a substantial reduction in CVD‐related mortality when their dietary magnesium intake was increased [31, 32, 33]. Hypertension, a well‐established risk factor for cardiovascular mortality [34, 35] is strongly associated with an increased risk of cardiovascular and cerebrovascular events. Magnesium contributes to cardiovascular health by regulating endothelial function, myocardial excitability, and vascular smooth muscle tone [36]. Therefore, increasing magnesium intake may serve as an effective nutritional therapeutic strategy to reduce hypertension and cardiovascular mortality in individuals with CNP. Notably, higher dietary magnesium intake was not significantly associated with a lower risk of cardiovascular mortality in individuals with CNP aged 60 years and older. This observation is thought to be related to age‐related declines in intestinal magnesium absorption capacity, suggesting that elderly individuals with CNP may require non‐dietary interventions. However, there is currently no clear scientific evidence to validate this hypothesis.

Our findings further address the inconsistent evidence regarding the role of magnesium in CNP as mentioned in the Introduction. Studies by Okada et al. [5]. and Bagheri et al. [6]. suggested that magnesium may alleviate muscle spasms and inflammation in individuals with CNP, whereas Igwe et al. found no significant association between serum magnesium levels and CNP severity. The present study extends these observations by linking long‐term dietary magnesium intake to mortality outcomes rather than just pain severity, which may help explain the conflicting conclusions in previous research. Furthermore, our results are consistent with population‐based studies indicating that higher magnesium intake is associated with a lower risk of CVD and reduced inflammation in chronic musculoskeletal disorders. These comparisons highlight the novelty of our dose‐response and threshold analyses, which have not been fully explored in prior CNP‐focused research.

Additionally, this study identified a non‐linear, monotonically decreasing association between dietary magnesium intake and cancer mortality among individuals with CNP. Specifically, there was a consistent yet irregular linear trend of reduced cancer mortality risk with increasing dietary magnesium intake. Threshold effect analysis was employed to determine the critical threshold for magnesium intake, revealing that individuals with CNP experienced a significant reduction in cancer‐related mortality risk when their dietary magnesium intake exceeded 0.5181 g/day. This study holds important practical implications for formulating therapeutic dietary advice and managing mortality risk in this population. It suggests that increasing dietary magnesium intake to at least 0.5181 g/day may exert a protective effect against cancer‐related mortality among individuals with CNP.

To ensure clinical safety, it is crucial to define the upper limit of dietary magnesium intake to prevent hypermagnesemia, which may lead to muscle weakness, cardiac arrhythmia, or renal impairment. According to the Dietary Reference Intakes for adults, the UL of magnesium from all sources (including food and supplements) is 700 mg/day (0.70 g/d). Notably, the protective threshold identified in this study (0.5181 g/d) is well below this safe upper limit, indicating that the recommended magnesium intake level is safe for most individuals with CNP. For individuals with impaired renal function, it is advisable to reduce magnesium intake and monitor serum magnesium levels regularly to avoid adverse effects.

Additionally, mediation analysis confirmed the crucial role of CRP in the association between dietary magnesium intake and mortality. CRP is an inflammatory biomarker synthesized by the liver, which reliably reflects the degree of systemic inflammation and tissue damage. By reducing log‐transformed CRP levels, dietary magnesium indirectly lowers the risk of CVD and all‐cause mortality in individuals with CNP, which is fully consistent with the findings of previous studies. Mechanistically, magnesium acts as a natural calcium antagonist, competitively inhibiting calcium influx and regulating intracellular calcium signaling. Excessive intracellular calcium promotes the phosphorylation and NF‑κB — a key transcription factor that drives the expression of pro‐inflammatory genes, including TNF‑α and IL‑6. By inhibiting calcium‐dependent activation of NF‑κB, magnesium suppresses the release of TNF‑α, IL‑6, and other downstream inflammatory mediators, thereby alleviating chronic low‐grade inflammation (referred to as “inflammaging”) — a critical driver of mortality in chronic musculoskeletal and age‐related disorders. From a pathophysiological perspective, vascular endothelial cells are continuously damaged by chronic inflammation during the progression of CNP, which gradually leads to the formation of atherosclerotic plaques and impairs their stability. Plaque rupture and thrombosis can trigger myocardial infarction and stroke, the primary causes of cardiovascular mortality in individuals with CNP [37, 38]. Chronic inflammation also impairs the immune system, liver, kidneys, and other organ systems, potentially leading to septic shock or multiple organ failure, which indirectly increases the risk of all‐cause mortality [39, 40]. Dietary magnesium specifically interrupts this critical link in the pathogenic chain, reducing systemic inflammation by lowering CRP levels. Furthermore, adjusting for CRP in the fully adjusted Cox regression model did not eliminate the protective association between magnesium intake and mortality. This finding supports the existence of two distinct mechanisms: CRP‐mediated anti‐inflammatory effects and additional non‐inflammatory pathways through which magnesium exerts its protective effects.

However, several limitations of this study should be acknowledged. First, certain potential confounders including medication use and imaging data were not adjusted for in the analysis, which may have influenced the observed associations between dietary magnesium intake and mortality outcomes. Second, dietary magnesium intake was assessed using 24 h dietary recalls, a method inherently prone to recall bias and may not fully capture long‐term nutritional patterns. To enhance the reliability of the data, we used the average of two 24 h dietary recalls as the baseline measure of magnesium intake. Third, this study relied on self‐reported questionnaire data from the NHANES database, which may introduce reporting biases or measurement errors. Our findings suggest that optimizing dietary magnesium intake may be a potential strategy for improving the long‐term prognosis of individuals with CNP, though randomized controlled trials are needed to confirm these clinical benefits.

## 5 Conclusion

In summary, this study confirmed a significant negative correlation between dietary magnesium intake and mortality in individuals with CNP, with higher dietary magnesium intake associated with reduced mortality risk. Furthermore, our findings suggest that increased dietary magnesium intake decreases all‐cause and CVD mortality in individuals with CNP, mediated by CRP. Meanwhile, we also propose incorporating CRP into routine CNP assessments. Notably, these findings are specific to individuals with CNP and cannot be generalized to the general healthy population. Additionally, the observed beneficial effects are associated with dietary magnesium derived from whole food sources (e.g., nuts, leafy green vegetables), rather than high‐dose magnesium supplements—these supplements carry a risk of hypermagnesemia, particularly in individuals with renal impairment. Future studies should investigate longitudinal changes in dietary magnesium intake and mortality in individuals with CNP to clarify the causal relationship in the CRP‐mediated pathway.

## Funding

This work was supported by Construction Project of Key Traditional Chinese Medicine Specialty (Orthopedics and Traumatology) in Chaoyang District, Beijing., National Natural Science Foundation of China (No. 82205151), China Academy of Chinese Medical Sciences Basic Scientific Research Business Expenses Outstanding Young Scientific and Technological Personnel Training Project (No. ZZ14‐YQ‐022), and National Key R&D Program of China (No. 2024YFC3507401).

## Ethics Statement

This study was conducted in accordance with the Declaration of Helsinki. The NHANES database was approved by the National Center for Health Statistics (NCHS) Ethics Review Committee. All participants provided written informed consent. This is a retrospective observational study based on a public database. It is not a clinical trial, and no clinical study registration number is required.

## Disclosure

The authors declare that the research was conducted in the absence of any commercial or financial relationships that could be construed as a potential conflict of interest.

## Conflicts of Interest

The authors declare no conflicts of interest.

## Data Availability

The data that support the findings of this study are available from the corresponding author upon reasonable request.

## References

[1] X. Zhang , C. Wacharasin , J. C. Dallas , and M. Ye , “Factors Predicting Quality of Life Among Patients With Cervical Spondylosis Undergoing Acupuncture in China: A Cross‐sectional Study,” Acupuncture in Medicine 43, no. 5 (2025): 256–264. 41045139 10.1177/09645284251379944

[2] W. C. He and J. Luo , “Comment on: Self‐stretching Exercises With Kinesio Taping for Management of Chronic Nonspecific Neck Pain in Taxi Drivers: A Single‐blind, Randomized Controlled Trial,” Complementary therapies in medicine 92 (2025): 103120. 40707105 10.1016/j.ctim.2024.103120

[3] T. Hirai , Y. Matsukura , S. Egawa , H. Onuma , and T. Yoshii , “Complications of Anterior and Posterior Surgery for Degenerative Cervical Myelopathy,” Journal of clinical orthopaedics and trauma 69 (2025): 103159. 40838087 10.1016/j.jcot.2025.103159 PMC12361775

[4] M. Lu , Y. Tang , X. Geng , C. Gu , Y. Zhao , and X. Chen , “MRI‐based Vertebral Bone Quality Score in Cervical Ossification of the Posterior Longitudinal Ligament‐A Comparison With Cervical Spondylotic Myelopathy Using Propensity Score Matching,” The spine journal: Official journal of the North American Spine Society 24, no. 7 (2024): 1153–1161. 38447872 10.1016/j.spinee.2024.02.015

[5] A. Okada , T. Kato , K. Okubo , Y. Kurokawa , and K. Koyama , “Early Recognition of Hypermagnesemia‐Induced Prolonged Muscle Relaxation and Delayed Arousal through Ionized Magnesium Measurement,” Cureus 17, no. 3 (2025): 79865. 10.7759/cureus.79865 PMC11955857 40166788

[6] G. R. Bagheri , J. Poursamimi , H. A. Anvari , S. R. Ghalenoo , and H. R. Ghaffari , “The Immune Base Therapy of Pain With Magnesium Sulfate on the Trigger Axis of the TNF‐α‐TRAF6‐NF‐κB and Its Inhibitor (miR‐146a‐5p) in Rats,” Iranian journal of allergy, asthma, and immunology 24, no. 2 (2025): 180–186. 40211498 10.18502/ijaai.v24i2.18146

[7] A. A. Igwe , O. K. Onyeso , I. Adandom , et al., “An Exploratory Cohort Study of Serum Estradiol, Testosterone, Osteoprotegerin, Interleukin‐6, Calcium, and Magnesium as Potential Biomarkers of Cervical Spondylosis,” Bulletin of Faculty of Physical Therapy 28, no. 1 (2023): 29–29.

[8] E. Bansal , S. Shende , S. Srivastava , and N. Tandon , “A Comparative Evaluation of Dexmedetomidine and Magnesium Sulfate During Awake fiber Optic Orotracheal Intubation in Patients Scheduled for Cervical Spine Surgeries: A Prospective Study,” Asian Journal of Medical Sciences 14, no. 3 (2023): 39–45.

[9] J. Ye , B. Miao , Y. Xiong , et al., “3D printed Porous Magnesium Metal Scaffolds With Bioactive Coating for Bone Defect Repair: Enhancing Angiogenesis and Osteogenesis,” Journal of Nanobiotechnology 23, no. 1 (2025): 160–160. 40033312 10.1186/s12951-025-03222-3 PMC11874660

[10] Z. Shi , J. Tuomilehto , M. Jugaitis , et al., “Association of Low Serum Magnesium With Diabetes and Hypertension: Findings From Qatar Biobank Study,” Diabetes Research and Clinical Practice 158 (2019): 107903, Human Nutrition Department CoHS, QU Health, Qatar University, Doha, Qatar, Qatar Metabolic Institute ED, Department of Medicine, Hamad Medical Corporation, Weill Cornell Medicine—Qatar D, Qatar. 31678625 10.1016/j.diabres.2019.107903

[11] K. Kostov , “Effects of Magnesium Deficiency on Mechanisms of Insulin Resistance in Type 2 Diabetes: Focusing on the Processes of Insulin Secretion and Signaling,” International Journal of Molecular Sciences 20, no. 6 (2019): 1351. 30889804 10.3390/ijms20061351 PMC6470576

[12] W. Mengyan , P. Jianhong , Y. Caili , Z. Wenyuan , C. Zicheng , and Z. Haibin , “Magnesium Intake and all‐cause Mortality After Stroke: A Cohort Study,” Nutrition journal 22, no. 1 (2023): 54–54. 37899441 10.1186/s12937-023-00886-1 PMC10614364

[13] E. Ilse , C. Esther , K. Iris , R. M. R. Rouwette , M. C. Geleijnse , and J. M. Geleijnse , “Dietary Magnesium and Risk of Cardiovascular and all‐cause Mortality After Myocardial Infarction: A Prospective Analysis in the Alpha Omega Cohort,” Frontiers in Cardiovascular Medicine 9 (2022): 936772–936772. 36035961 10.3389/fcvm.2022.936772 PMC9416912

[14] B. Lori , M. CM , and L. BV , “National Health and Nutrition Examination Survey: National Youth Fitness Survey Plan, Operations, and analysis,” Vital and Health Statistics Series 2, Data Evaluation and Methods Research 158 (2012): 1–24. 24709592

[15] J. PC , P. Nam , M. Michael , et al., “A Database of human Exposomes and Phenomes From the US National Health and Nutrition Examination Survey,” Scientific data 3, no. 1 (2016): 160096. 27779619 10.1038/sdata.2016.96 PMC5079122

[16] X. Bo , M. Guoliang , Y. Liu , et al., “Non‐linear Association of Atherogenic Index of Plasma With Bone Mineral Density a Cross‐sectional Study,” Lipids in Health and Disease 23, no. 1 (2024): 181–181. 38867213 10.1186/s12944-024-02180-3 PMC11167925

[17] F. Wu , M. Ferguson , A. Lando , and L. Verrill , “Human Foods Program USF, Administration D. Self‐Efficacy: The Key to Nutrition Facts Label Use—Theory‐Based Findings From the 2019 FDA Food Safety and Nutrition Survey (FSANS),” Journal of the Academy of Nutrition and Dietetics 12 (2025): 267–275. 10.1016/j.jand.2025.09.007 40953745

[18] E. A. Andrade and M. C. B. Galvão , “International Statistical Classification of Diseases and Related Health Problems (ICD‐11): From Its Origin to Its Use in Digital Systems,” Ciencia & saude coletiva 30 (2025): 1–9. 10.1590/1413-812320242911.01402024 40471591

[19] E. L. Honig , S. Kaveeshwar , and N. N. O'Hara , “Greater Socioeconomic Deprivation Predicts Worse Functional Status Two Years After Orthopaedic Surgery, but Not Magnitude of Change From Baseline,” Journal of orthopaedics 70 (2025): 33–38. 40225055 10.1016/j.jor.2025.03.022 PMC11984530

[20] X. Furong , E. J. Edwards , A. Alessandra , W. Lee , and G. G. Greene , “The Relationship of Physical Activity and Dietary Quality and Diabetes Prevalence in US Adults: Findings From NHANES 2011–2018,” Nutrients 14, no. 16 (2022): 3324–3324. 36014830 10.3390/nu14163324 PMC9414710

[21] L. Yuting , Y. Xiaojing , Z. Qiutong , et al., “The association of periodontal disease and oral health with hypertension: A nationwide study in China,” BMC Public Health [Electronic Resource] 23, no. 1 (2023): 1122–1122. 37308938 10.1186/s12889-023-16012-z PMC10262359

[22] A. MMD , I. N. dS , R. Virgílio , et al., “Metabolic Equivalent of Task (METs) Thresholds as an Indicator of Physical Activity Intensity,” PLoS ONE 13, no. 7 (2018): 0200701. 10.1371/journal.pone.0200701 PMC6053180 30024953

[23] S. Y. Kim , “Physical Activity, Weekend Catch‐up Sleep, and Depressive Symptoms: Mediating Effects of High‐sensitivity C‐reactive Protein,” Sleep Medicine 138, no. S (2026): 108417–108417. 10.1016/j.jad.2025.119452 40398607

[24] P. Sakhamuru , M. Nayeni , R. Nazari , K. Syed , and K. Miller , “An Uncommon Presentation of Crowned Dens Syndrome without Systemic Inflammation,” Cureus 17, no. 5 (2025): e84853–e84853. 40568261 10.7759/cureus.84853 PMC12194727

[25] E. MM and A. CJ , “Magnesium Chemistry and Biochemistry,” Biometals: An international journal on the role of metal ions in biology, biochemistry, and medicine 15, no. 3 (2002): 203–210. 12206387 10.1023/a:1016058229972

[26] M. Amjad , S. S. U. Rehman , G. Fatima , M. Ikram , and S. Ghafoor , “Comparative Effects of Isometric and Isotonic Global Neck Muscles Strengthening Exercise Programme on Pain,” The Journal of the Pakistan Medical Association 74, no. 10 (2024): 1843–1846. 39407381 10.47391/JPMA.11378

[27] S. Jabbari , Z. A. Zakaria , and S. Mohammadi , “Antinociceptive and Antineuropathic Effects of Trifolium Resupinatum L. on Formalin‐induced Nociception and Cervical Spinal Cord Hemi‐contusion: Underlying Mechanisms,” Journal of ethnopharmacology 337, no. P2 (2024): 118913. 39369921 10.1016/j.jep.2024.118913

[28] F. H. Nielsen , “Magnesium Deficiency and Increased Inflammation: Current Perspectives,” Journal of Inflammation Research 11 (2018): 25–34. 29403302 10.2147/JIR.S136742 PMC5783146

[29] B. Mario , B. Mario , and J. DL , “Magnesium Homeostasis and Aging,” Magnesium research 22, no. 4 (2009): 235–246. 20228001 10.1684/mrh.2009.0187

[30] C. W. Jerzy , L. Jerzy , D. Małgorzata , C. Anna , and B. W. Magnesium , “C‐reactive Protein, and Cortisol in Drug‐naïve Patients With Short Illness‐duration, First Episode Major Depressive Disorder: Possible Immunomodulatory Role for Magnesium,” Magnesium research 29, no. 4 (2016): 169–174. 27965189 10.1684/mrh.2016.0413

[31] H. Hedong , F. Xin , W. Xin , et al., “Dose‐response Relationship Between Dietary Magnesium Intake, Serum Magnesium Concentration and Risk of Hypertension: A Systematic Review and Meta‐analysis of Prospective Cohort Studies,” Nutrition journal 16, no. 1 (2017): 26. 28476161 10.1186/s12937-017-0247-4 PMC5420140

[32] X. Fang , C. Liang , M. Li , et al., “Dose‐response Relationship Between Dietary Magnesium Intake and Cardiovascular Mortality: A Systematic Review and Dose‐based Meta‐regression Analysis of Prospective Studies,” Journal of Trace Elements in Medicine and Biology 38 (2016): 64–73. 27053099 10.1016/j.jtemb.2016.03.014

[33] W. Wenjie , W. Xiaoyan , C. Shiling , et al., “Dietary Antioxidant Indices in Relation to all‐Cause and Cause‐Specific Mortality among Adults with Diabetes: A Prospective Cohort Study,” Frontiers in Nutrition 9 (2022): 849727–849727. 35600816 10.3389/fnut.2022.849727 PMC9116439

[34] Y. Menglin , Z. Huimin , and L. Rong , “Meta‐analysis on the Risk of all‐cause Mortality and Cardiovascular Death in the Early Stage of Hypertension,” Pakistan journal of pharmaceutical sciences 29 (2016): 1343–1351. 27592484

[35] Y. Huang , L. Su , X. Cai , et al., “Association of all‐cause and Cardiovascular Mortality With Prehypertension: A Meta‐analysis,” American Heart Journal 167, no. 2 (2014): 161. 10.1016/j.ahj.2013.10.023 24439976

[36] K. Dhaval , V. Krishnaswami , K. Sahil , A. SD , and H. FW , “Role of Magnesium in Cardiovascular Diseases,” Cardiology in review 22, no. 4 (2014): 182–192. 24896250 10.1097/CRD.0000000000000003

[37] L. Meng , L. Jinjin , Z. Yang , X. Fengying , W. Eerdun , and L. Xiaofeng , “The High‐sensitivity C‐reactive Protein to Lymphocyte Ratio Is Associated With all‐cause and Cardiovascular Mortality in U.S. adults: A Cohort Study From NHANES,” Medicine 104, no. 38: e44645–e44645. 40988208 10.1097/MD.0000000000044645 PMC12459476

[38] Y. Sun , Y. Guo , S. Ma , et al., “Association of C‐reactive Protein‐triglyceride Glucose Index With the Incidence and Mortality of Cardiovascular Disease: A Retrospective Cohort Study,” Cardiovascular Diabetology 24, no. 1 (2025): 313–313. 40750895 10.1186/s12933-025-02835-0 PMC12317521

[39] Z. Wang , Q. Zheng , X. Chen , and H. Wang , “Exploring the Association Between Cumulative hs‐CRP and all‐cause Mortality, With Consideration of Cardiometabolic Mediators in Middle‐aged and Elderly Adults: Insights From Observational Study: Hs‐CRP and Overall Mortality Risk,” J Archives of Gerontology and Geriatrics 135 (2025): 105861–105861. 10.1016/j.archger.2025.105861 40354683

[40] J. Zhang , Y. Lin , J. Zeng , et al., “The C‐reactive Protein (CRP)‐albumin‐lymphocyte (CALLY) Index Exhibits an L‐shaped Association With all‐cause Mortality in Rheumatoid Arthritis Patients: A Retrospective Cohort Study,” BMC Rheumatology 9, no. 1 (2025): 47–47. 40264172 10.1186/s41927-025-00499-7 PMC12013003

